# To My Mallet.—Apostrophe!!

**Published:** 1860-06

**Authors:** W. H. Atkinson


### TO MY MALLET.—APOSTROPHE!!

BY W. H. ATKINSON.

Good morning, old comrad, I hail thee with joy
And thank thee sincerely for thy *timely* aid:
Indispensible helper and not a mere *toy*,
The tribute that's due thee, though late, shall be paid.

I acknowledge thy (*a*) "Merit" and value thee (*b*) "More"
As thy power looms up the better we know thee—
Salvation to me in the dark days of yore
I gladly announce it that others may SEE.

Poor old-round-pointed Pluggers the *best* to be had,
Were the means with which Dentists then "stuffed the old stumps.'
Without thee! I *too* would have been a luckless poor Lad
Hadst thou not redeemed me by (*c*) "*Her*" gentle thumps,

That sent the pure (*d*) "Bulls' Foil" down to the bottom
Of all the *small* places we dared to attack
Her force through the rushing in current "Per Saltem"
Securely induced it there closely to Pack—

And *stay there till this time* (as eyes can aver)
Despite all my ignorant innocent youth
Because thou wert with me the evil to blur
To lay a *Foundation* for thee and for Truth.

My constant companion for twenty-two years,
I acknowledge I'm tardy in *writing* thy fame,
Though oft I in using thee saved many tears
To myself not alone but to Patient the same.

But in *speaking* of thee to Pupil or brother
I have not shunned the whole council to declare,'
Be it to this one, to that, or the other,
The way of success for them, through thee to prepare.

Some thought *me* a *bore* the *Mallet* a *notion*—
Some who ne'er tried, "could do better without thee"—
"Than I could do *with thee*" (propelled by the motion
Of Pupil intelligent helper or me.)

"And" insert in a day (for a traveling Patient)
Full forty-five fillings as hard as could be,
And pocket the cash for work which is latent-
Disease, when compared to the work done by thee!

Then the Mallett—Pure gold—in Crystals or Foil,
A Head—well-informed, a Heart honest and free;
A Hand—well-instructed, acquainted with toil,
Combine them in action and *work* you will see.

Yes, *work* that will stand to the test of old time,
Whose finger disintegrates Rock, Shell and Tree—
No changes of season location or Clime
Have wrought, *can* work such changes in work done by thee.

All patients "prefer the Mallet to" "pushing,"
As all who have testified to me agree;
One only exception, who chose the rushing
Of hand force in packing, my Mallet, to thee—

Not only the patient but all who well use it
Know and acknowledge pre-eminence ever
(*e*) Try it Profession before you abuse it
And you'll be well paid for your honest endeavor.

Try it on Soft-Foil, Adhesive or stubborn,
Try it on Crystals of "Taft & Watt" or "Watts"
"Serrated pluggers" or those that are "point-worn"
Will pack it in "Cavities" "Fissures" or "Slots."

Try it for *safety* to tooth and mouth-tissue,
Try it for *ease* to thyself and thy patient—
Give it fair trial 'twill prove in the issue
Econ'my of power both active and latent.

Try it for "any thing" *fit* to fill teeth with.
(Osteoplastics, Amalgams and putty
Need no such inducement—lack-skill to deal with
That lays not within the pathway of duty.)

Be honest, patient—*persistently* use it;
A harvest 'twill bring you most rich rare and ripe;
Once well you *know* it you can not refuse it
As helper, as saver, economy's type.

---

(*a*) Dr. E. Merrit, of Pittsburgh, Penn., was the first one that I ever knew to use the Mallet.

(*b*) J. G. Moore of Painsville used the Mallet for me in 1847 or 1848.

(*c*) My wife was my first and best assistant, and my only "help" for several years.

(*d*) Bulls of Philadelphia was the first beater whose foil I ever used.

(*e*) Apostrophe to the Profession in favor of the Mallet.

